# Pentalogy of Fallot and cardiac paraganglioma: a case report

**DOI:** 10.1186/1757-1626-2-9392

**Published:** 2009-12-23

**Authors:** Sushma Kashinath Gabhane, Nitin M Gangane, Riti T Sinha

**Affiliations:** 1 Department of Pathology, Mahatma Gandhi Institute of Medical Sciences, Sevagram, Wardha - 442102, Maharashtra, India

## Abstract

Primary cardiac tumors are rare. Of these the majorities are benign and about 75% are atrial myxomas. One of the rarest tumors affecting the heart is a cardiac paraganglioma. We report an unusual case of a left ventricular paraganglioma discovered during autopsy in a 22-year female patient, a case of sudden death. This patient also had coexistent Pentalogy of Fallots along with transposition of pulmonary trunk to the left ventricle, a very rare congenital cyanotic heart disease. Chronic hypoxia due to congenital cyanotic heart disease is supposed to be the cause of development of paraganglioma in heart in these patients.

## Case presentation

A 22-year caucaesian female patient, Indian by nationality was brought in emergency to casualty with the history of cyanotic spells and breathlessness. She succumbed to sudden death within two hours of admission to the hospital. The relatives gave history of some congenital heart disease, but no previous medical records were available. Being the case of sudden death an autopsy examination was performed.

**Figure 1:**
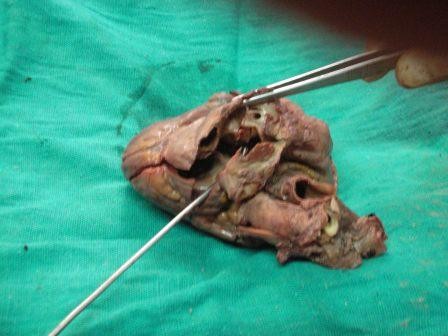
**Gross photograph of heart showing atrial septal defect**.

**Figure 2:**
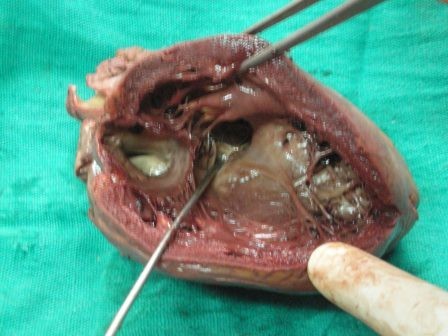
**Gross photograph of heart showing ventricular septal defect**.

**Figure 3:**
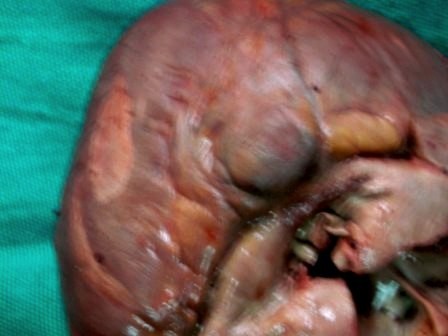
**Photograph showing tumor mass on posterior aspect of left Ventricle just below the atrioventricular groove**.

**Figure 4:**
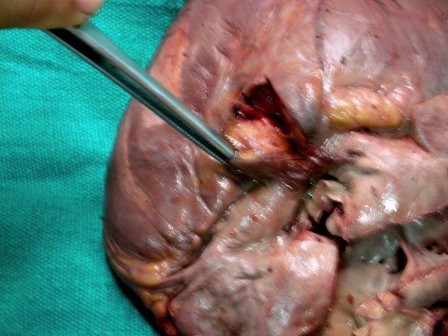
**Photograph showing a well defined, intramural, gray white, tumor mass on cut surface**.

On external examination the patient was thin built, cachectic with presence of cyanosis on lips, mucous membranes, nail bed, palms and soles. Internal examination revealed congestion of all viscera. The heart appeared enlarged weighing 400 gm. On cutting open it revealed an atrial septal defect of 1 cm diameter (Figure 1), a ventricular septal defect of 1.8 cm diameter in membranous part of septum (Figure 2), over-riding of aorta on VSD, dilatation and hypertrophy of right ventricle, left ventricular hypertrophy and origin of pulmonary trunk from left ventricle. Pulmonary trunk was dilated. Apart from these congenital defects the heart also showed a tumor mass on posterior aspect of left ventricle just below the atrioventricular groove (Figure 3). Cut surface of this mass was well defined, encapsulated, intramural, gray-white, firm, of 2 cm diameter (Figure 4). Sections from the tumor mass and other routine sections of heart were taken for microscopic examination. Lungs were grossly heavy, wet, and congested. Cut surface showed oozing of frothy fluid from smaller bronchi and alveoli. Liver and kidney showed congestion.

**Figure 5:**
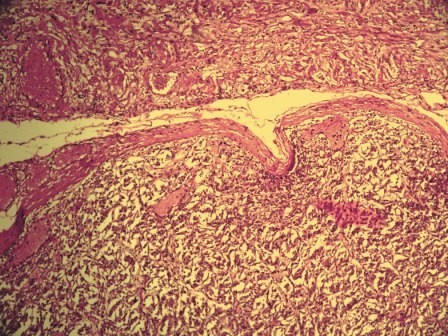
**Photomicrograph showing encapsulated tumor with 'Zellenballen pattern' (H&E, 40×)**.

**Figure 6:**
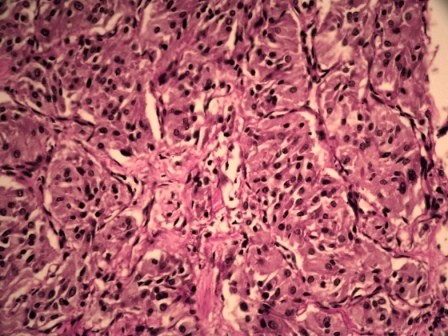
**Photomicrograph showing nests of polygonal cells with eosinophilic cytoplasm and round regular nuclei with fine chromatin (H&E, 400×)**.

Microscopic examination of sections from heart showed unremarkable myocardium and coronary blood vessels. Tumor mass in left ventricle was highly cellular and comprised of nests of polygonal to oval cells with eosinophilic cytoplasm. The nuclei were round regular with fine chromatin. The nests of cells were surrounded by sustentacular cells and separated delicate fibrovascular stroma giving the appearance of 'Zellenballen pattern'. All these features suggested the diagnosis of a cardiac paraganglioma. (Figure 5 and figure 6)

Sections from lung showed pulmonary edema, interstitial inflammation and congestion. Sections from kidney and liver showed congestion.

## Discussion

There are no more than 50 previously reported cases of primary cardiac paraganglioma in the world literature and only a small proportion of these involve the right side of the heart. The antemortem diagnosis of such a case is a difficult one [1].

The incidence of congenital heart disease (CHD) among all live births in India has been reported to be 0.5-0.8%. Tetralogy of Fallot is the second commonest congenital heart disease seen in almost 17.86% of cases after Ventricular septal disease (36.73%). Pentalogy of Fallots, although rare but contributes for 3.7% of all CHD [2]. On the other hand, paraganglioma occurs in approximately 0.1% of the population, and an even lower rate, approximately 2 cases/1,000,000 people/year [3]. Both diseases are well known but relatively rare, and thus the probability of their occurring co-existently is extremely low.

Paraganglioma may arise in numerous locations like nasopharynx, larynx, orbit, gallbladder, duodenum, kidney, urinary bladder and heart [3]. Cardiac paraganglioma is one of the rarest forms of paraganglioma, with only 50 cases reported in the literature. Most cases have occurred in women with average age of 45 years. The tumor occurs primarily in the left atrium or in the interventricular groove at the aortic root and commonly gives rise to hypertensive symptoms. The lesions are histologically and immunohistochemically quite similar to other forms of paraganglioma. Two of the 50 cases reported in literature had developed metastasis [1,3].

Intrathoracic paragangliomas are mostly located in the posterior mediastinum. However, these tumors can also occur in close association with the left atrial or left ventricular epicardium, where they are thought to arise from sympathetic fibers to the heart or from ectopic chromaffin cells. Tumors in any of these locations may secrete catecholamines and therefore can be associated with signs and symptoms characteristic of pheochromocytoma [4,5].

Maxey et al [6] have reported a biatrial primary cardiac paraganglioma discovered during workup for palpitations and fatigue. Turley et al [7] and Jimenz et al [8] have also reported primary cardiac paraganglioma in left atrium in 56-year and 59-year old man respectively. Geiser et al [9] have described a presence of paraganglioma located in the atrio-ventricular sulcus also involving the trunk and bifurcation branches of the left coronary artery which they detected on post-mortem examination of a 26-year old man.

Tetralogy of Fallot is one of the most common congenital cardiac defects causing cyanosis. It is charecterised by biventricular origin of the aorta above a large VSD, obstruction to pulmonary blood flow, and right ventricular hypertrophy. Tetralogy when associated with ASD is called Pentalogy of Fallot, and is not distinguishable clinically [10]. Pentalogy of Fallot and transposition of pulmonary trunk to the left ventricle is a very rare combination which was seen in the present case.

Nissenblatt [11] described the development of a carotid body tumor in a young woman with hypoplastic right heart syndrome and chronic cyanosis. He attributed the development of the chemodectoma to the physiologic stimulus of chronic hypoxia. Review of the literature disclosed 59 previously reported cases of hypoxia associated with endocrine tumors. The hypoxic state stimulates catecholamine secretion from the adrenal medulla, and chronic endocrine hyper-reactivity may lead to hyperplasia and neoplasia [12].

Lack [13] and Chadid and Jao [14] also confirmed the similar findings. They found on ultrastructural and tissue culture studies that all the structures present in the normal carotid body are present as exaggerated counterparts in the tumors. This association clarified the increased incidence of both hyperplasia and neoplastic transformation in response to the common stimulus of chronic hypoxia. Recent literature has also proposed the development of carotid body tumors as a response to the chronic hypoxia in patients with cyanotic congenital heart disease [11,13,14].

The combination of paraganglioma with Tetralogy of Fallot or cyanotic congenital heart disease is rare; however, these might be related through chronic hypoxia and/or gene abnormalities. The presence of paraganglioma worsens the hemodynamic state in patients with congenital heart disease regardless of whether radical surgery for congenital heart disease had been performed [15].

In conclusion, we report a very rare and unusual case of cardiac paraganglioma associated with Pentalogy of Fallot and transposition of pulmonary trunk to the left ventricle. The development of paraganglioma in heart may be due of chronic hypoxia because of cyanotic congenital heart disease. The presence of both the pathologies together worsen the patients condition and turned out to be fatal in some cases as in this case. Hence, patients of cyanotic congenital heart diseases must be monitored regularly for early detection of development of such tumors.

## Abbreviations

ASD: atrial septal defect; CHD: congenital heart disease; VSD: ventricular septal defect.

## Consent

A written consent has been obtained from the first degree relatives of this patient (as the findings were seen on autopsy). No personal identifiers are used in this case report.

## Competing interests

The authors declare that they have no competing interests.

## Authors' contributions

SG done the autopsy in this case, confirmed the diagnosis of the case, and wrote the first draft of the manuscript. NG confirmed the diagnosis of the case, and critically reviewed the manuscript. RS contributed in searching the literature and photography for the case.

## References

[B1] Kennelly R Aziz R Toner M Young V Right atrial paraganglioma: an unusual primary cardiac tumor Eur J Cardiothorac Surg 2008 33 6 1150 1152 10.1016/j.ejcts.2008.02.031 18406162

[B2] Behrman RE Kliegman RM Jenson HB Behrman RE, Kliegman RM, Jenson HB From Congenital heart disease Nelson textbook of Pediatrics 2000 16 Philadelphia: Harcourt Asia Pvt Ltd 1362 63

[B3] Enzinger FM Weiss SW Enzinger FM, Weiss SW From Paraganglioma Enzinger and Weiss Soft Tissue Tumors 1995 3 London: Mosby 965 990

[B4] David TE Lenkei SC Marquez-Julio A Goldberg JA Meldrum DA Pheochromocytoma of the heart Ann Thorac Surg 1986 41 98 100 3942440 10.1016/s0003-4975(10)64507-9

[B5] Sabatine MS Carlucci WS Schoen FJ Zipes DP, Libby P, Bonow RO, Braunwald E Primary tumors of the heart Braunwald's heart disease. A Textbook Of Cardiovascular Medicine 2005 7 Philadelphia: Elsevier Saunders 1741 1756

[B6] Maxey TS Grow P Morris CD Patton KT Guyton RA Biatrial primary cardiac paraganglioma: a rare finding Cardiovasc Pathol 2007 16 3 179 182 10.1016/j.carpath.2006.11.002 17502248

[B7] Turley AJ Hunter S Stewart MJ A cardiac paraganglioma presenting with atypical chest pain Eur J Cardiothorac Surg 2005 28 2 352 354 10.1016/j.ejcts.2005.04.038 15990328

[B8] Jimenz JF Warren ET Shroff RK Stolz GA Primary cardiac paraganglioma J Ark Med Soc 2005 101 12 362 364 15948504

[B9] Geisler F Barth G Jaeck D Pflumio F Tongio J Bellocq JP Steib A Aprosio N Batzenschlager A A case of pheochromocytoma with cardiac localization. Review of the literature Presse Med 1985 14 18 1024 1026 3158947

[B10] Fread MD William H Plauth B Alexander RW, Schilant RC, Fuster V, O'Rourlee RA, Roberts R, Sonnenblick EH From The pathology, pathophysiology, recognition and treatment of congenital heart disease Hurst's the heart, arteries and veins 1998 2 9 New York: McGraw Hill 1925 2030

[B11] Nissenblatt MJ Cynotic heart disease:"low altitude" risk for carotid body tumor? Johns Hopkins Med J 1978 142 18 21 625092

[B12] Deanfield JE Gersh BJ Warnes CA Douglas MD Alexander RW, Schilant RC, Fuster V, O'Rourlee RA, Roberts R, Sonnenblick EH Congenital heart disease in adults Hurst's the heart, arteries and veins 1998 2 9 New York: McGraw Hill 1925 2030

[B13] Lack EE Carotid body hypertrophy in patients with cystic fibrosis and cyanotic congenital heart disease Hum Pathol 1976 8 39 47 10.1016/S0046-8177(77)80064-6 844853

[B14] Chadid A Jao W Hereditary tumors of the carotid bodies and chronic obstructive pulmonary disease Cancer 1974 33 1635 1641 10.1002/1097-0142(197406)33:6<1635::AID-CNCR2820330625>3.0.CO;2-J 4366403

[B15] Kita Toshihiro Imamura Takuroh Date Haruhiko Kitamura Kazuo Moriguchi Sayaka Sato Yuichiro Asada Yujiro Eto Tanenao Two cases of pheochromocytoma associated with tetralogy of Fallot Hypertens Res 2003 26 433 437 10.1291/hypres.26.433 12887136

